# Imaging of Glutamate Concentration in Sturge-Weber Syndrome

**DOI:** 10.15844/pedneurbriefs-31-1-2

**Published:** 2017-01

**Authors:** Tracy S. Gertler, Cynthia V. Stack

**Affiliations:** 1 Division of Neurology, Ann & Robert H. Lurie Children’s Hospital of Chicago, Chicago, IL; and Departments of Pediatrics and Neurology, Northwestern University Feinberg School of Medicine, Chicago, IL

**Keywords:** Sturge-Weber Syndrome (SWS), Proton Magnetic Resonance Spectroscopic Imaging (MRSI), Glutamate

## Abstract

Investigators from Wayne State University studied a cohort of children with Sturge-Weber syndrome (SWS) and epilepsy using both glucose-based positron emission tomography (FDG-PET) to evaluate metabolic activity and proton magnetic resonance spectroscopic imaging (MRSI) to evaluate glutamate turnover.

Investigators from Wayne State University studied a cohort of children with Sturge-Weber syndrome (SWS) and epilepsy using both glucose-based positron emission tomography (FDG-PET) to evaluate metabolic activity and proton magnetic resonance spectroscopic imaging (MRSI) to evaluate glutamate turnover. The contextual goal of this study is development of a biomarker for intractable epilepsy in a high-risk population to facilitate stratification towards an early surgical work-up. Seizure frequency was graded at enrollment and one year later (or upon surgical resection). Both proton MRSI and FDG-PET were performed under sedation in parallel with contrasted 3T MRI. [1]

COMMENTARY. SWS is a sporadic neurocutaneous syndrome characterized by facial capillary malformation (port-wine stain; PWS) and leptomeningeal angiomatosis, now attributable to a somatic activating mutation of the guanine nucleotide-binding protein alpha (GNAQ) [2]. While epilepsy in SWS is controlled by a single AED in up to 70% of cases, intractable epilepsy beginning in infancy is clearly correlated with intellectual disability and functional impairment [3]. However, comparable to other types of early-onset, medically-intractable, focal epilepsy, surgical resection may offer seizure-freedom and cognitive improvement. Thus, a key question in the field is how to gauge candidacy for epilepsy surgery in SWS patients in a timely fashion.

Previous attempts at risk stratification of SWS patients have relied on mapping the dermatologic distribution of the PWS [4]; as defined by embryonic craniofacial patterns rather than trigeminal innervation, frontonasal PWS involvement was associated with higher risk of SWS severity. A parallel publication to the current study correlates the presence of bilateral PWS and developmental venous anomalies (DVA) with increased epilepsy risk, though no correlation was seen with involvement of specific embryonic facial placodes [5]. Indeed, in spite of consistent neuropathological findings of dystrophic vasculature, gliosis, and atrophy in resected tissue from SWS patients who underwent epilepsy surgery, MRI findings alone have been difficult to correlate with disease severity [6].

Taken in context, the current study offers a functional neuroimaging correlate (i.e. glutamate metabolism) which may portend severe epilepsy and thus suggest early surgical resection. Strengths of this study include parallel comparison of multiple imaging modalities involved in a surgical epilepsy work-up, as well as a well-defined patient population (unilateral SWS in pediatric patients with early-onset epilepsy) and clinical follow-up as far as one year after imaging. Size of the study population is an obvious limitation. As all data were normalized to the contralateral hemisphere and EEG was not performed, abnormal glutamate metabolism in the contralateral hemisphere is a potential confound. Imaging data from patients with confirmed SWS without epilepsy (or prior to seizure onset) were not available for comparison. In sum, this study suggests an important adjunct method of early evaluation of SWS patients who may benefit from epilepsy surgery.
